# Time to rethink academic publishing: the peer reviewer crisis

**DOI:** 10.1128/mbio.01091-23

**Published:** 2023-11-17

**Authors:** Carolina Tropini, B. Brett Finlay, Mark Nichter, Melissa K. Melby, Jessica L. Metcalf, Maria Gloria Dominguez-Bello, Liping Zhao, Margaret J. McFall-Ngai, Naama Geva-Zatorsky, Katherine R. Amato, Eduardo A. Undurraga, Hendrik N. Poinar, Jack A. Gilbert

**Affiliations:** 1 Humans and the Microbiome Program, Canadian Institute for Advanced Research, Toronto, Ontario, Canada; 2 Department of Microbiology and Immunology, University of British Columbia, Vancouver, British Columbia, Canada; 3 School of Biomedical Engineering, University of British Columbia, Vancouver, British Columbia, Canada; 4 Department of Biochemistry and Microbiology and the Michael Smith Laboratories, University of British Columbia, Vancouver, British Columbia, Canada; 5 School of Anthropology, University of Arizona, Tucson, Arizona, USA; 6 Department of Anthropology, University of Delaware, Newark, Delaware, USA; 7 Department of Animal Sciences, Colorado State University, Fort Collins, Colorado, USA; 8 Department of Biochemistry and Microbiology, Rutgers University, New Brunswick, New Jersey, USA; 9 Department of Anthropolog, Rutgers University, New Brunswick, New Jersey, USA; 10 Carnegie Institution for Science, California Institute of Technology, Pasadena, California, USA; 11 Department of Cell Biology and Cancer Science, Rappaport Faculty of Medicine, Technion-Israel Institute of Technology, Rappaport Technion Integrated Cancer Center (RTICC), Haifa, Israel; 12 Department of Anthropology, Northwestern University, Evanston, Illinois, USA; 13 Escuela de Gobierno, Pontificia Universidad Católica de Chile, Santiago, Región Metropolitana, Chile; 14 Research Center for Integrated Disaster Risk Management (CIGIDEN), Santiago, Región Metropolitana, Chile; 15 Departments of Anthropology and Biochemistry, McMaster University, Hamilton, Ontario, Canada; 16 Department of Pediatrics, Scripps Institution of Oceanography, University of California San Diego, La Jolla, California, USA; Stanford University, Stanford, California, USA

**Keywords:** peer review, reviewer rewards, artificial intelligence, early career researcher, peer review crisis

## Abstract

There is concern that the time taken to publish academic papers in microbiological science has significantly increased in recent years. While the data do not specifically support this, evidence suggests that editors are having to invite more and more reviewers to identify those willing to perform peer review.

## EDITORIAL

Academic publishing has, and continues to be, the cornerstone of scientific communication. It facilitates the reporting of findings from the application of the scientific method. One component of this process is the timely review of a primary article by scientific peers who can rigorously assess the originality, validity, and significance of the science presented. Peer review, while imperfect, provides an effective way of ensuring that the science published in a journal for broad consumption has at least been assessed by people who have confirmed that the approach, analysis, and interpretation as presented is, to the best of their understanding, appropriate and accurate. The COVID-19 pandemic and subsequent vaccination program highlighted the value of ensuring rigorous scientific communication. In many countries, a large proportion of the public appeared to reject scientific findings published in the peer-reviewed literature, choosing instead to follow their own “research” to justify their rejection of masks or vaccines. Calls for abandoning pre-publishing peer review, and instead relying on post-publication peer review, ignore the fact that a minority of scientists can and will publish, either deliberately or through gross negligence, “findings” that support a narrative that can cause real harm (1).

However, even with the current peer review process, articles that present inaccurate or misleading findings do get published, resulting in press coverage on vaccines causing autism (2), arsenic “life” (3), or plague on the New York subway (4). How does this happen? Each of these articles was published in a “respectable” academic publishing outlet, and somehow peer review failed to detect the glaring errors in the design, analysis, and interpretation as presented. Peer review fails for a number of reasons, including (i) a lack of rigorous training in how to review scientific articles, which, while an ostensible part of our scientific training, is at best neglected and often completely ignored; (ii) a lack of visible reward for peer reviewing, not only fiscally due to the inherent nature of the voluntary process but also due to institutions that tend of expect peer review as part of career progress but do not value it outright; and (iii) pure exhaustion on behalf of the available pool of peer reviewers, who become overburdened by requests for their services from journals.

The last point is especially problematic when a particular scientific field becomes popular, as, for example, microbiome research has in the last 10 years. This popularity leads to a proliferation of journals that attempt to “cash in” on the demand by the community for places to publish their research as the breadth of investigation leads to ever more specific research niches, or the novelty of the research declines, leading to rejection of submissions at “high-impact” venues. Whether these journals are supporting not-for-profit, community-facing scientific societies or are just a profit vehicle for entrepreneurial for-profit companies, the result is that so many articles require peer review that the burden on the available scientists with appropriate expertise becomes extreme, leading to burnout and the collapse of interest in providing this service (5).

We hypothesized that the length of time it takes to publish a scientific article in a microbiology research journal had been increasing, especially since the pandemic, resulting from an increase in the difficulty of finding appropriate expert peers to review the submissions. To test this hypothesis, we acquired data on the time from submission to first decision for 49,052 articles published in nine American Society for Microbiology (ASM) journals between 2016 and 2022. In addition, we acquired data over the same period that represented the number of peer reviewers that were contacted to review these articles during the assessment process.

**Fig 1:**
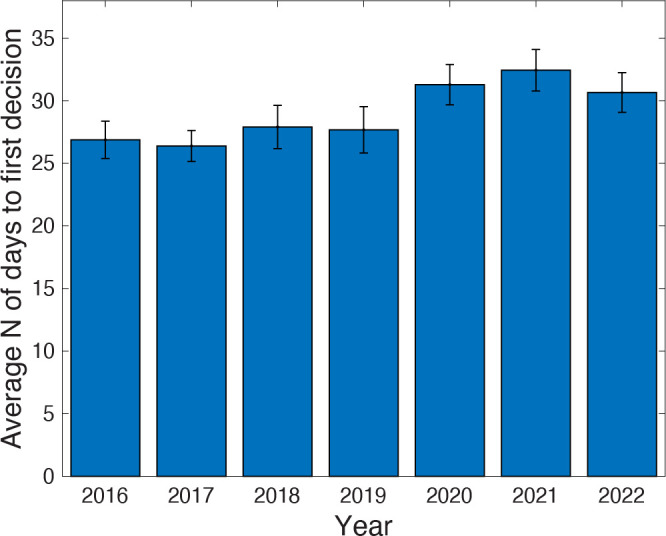
The average number of days from receipt to first decision (this includes the time from completion of the final review until the editor sent the decision letter) that a submission spent in peer review prior to publication across nine ASM journals. Error bars indicate standard error.

**Fig 2:**
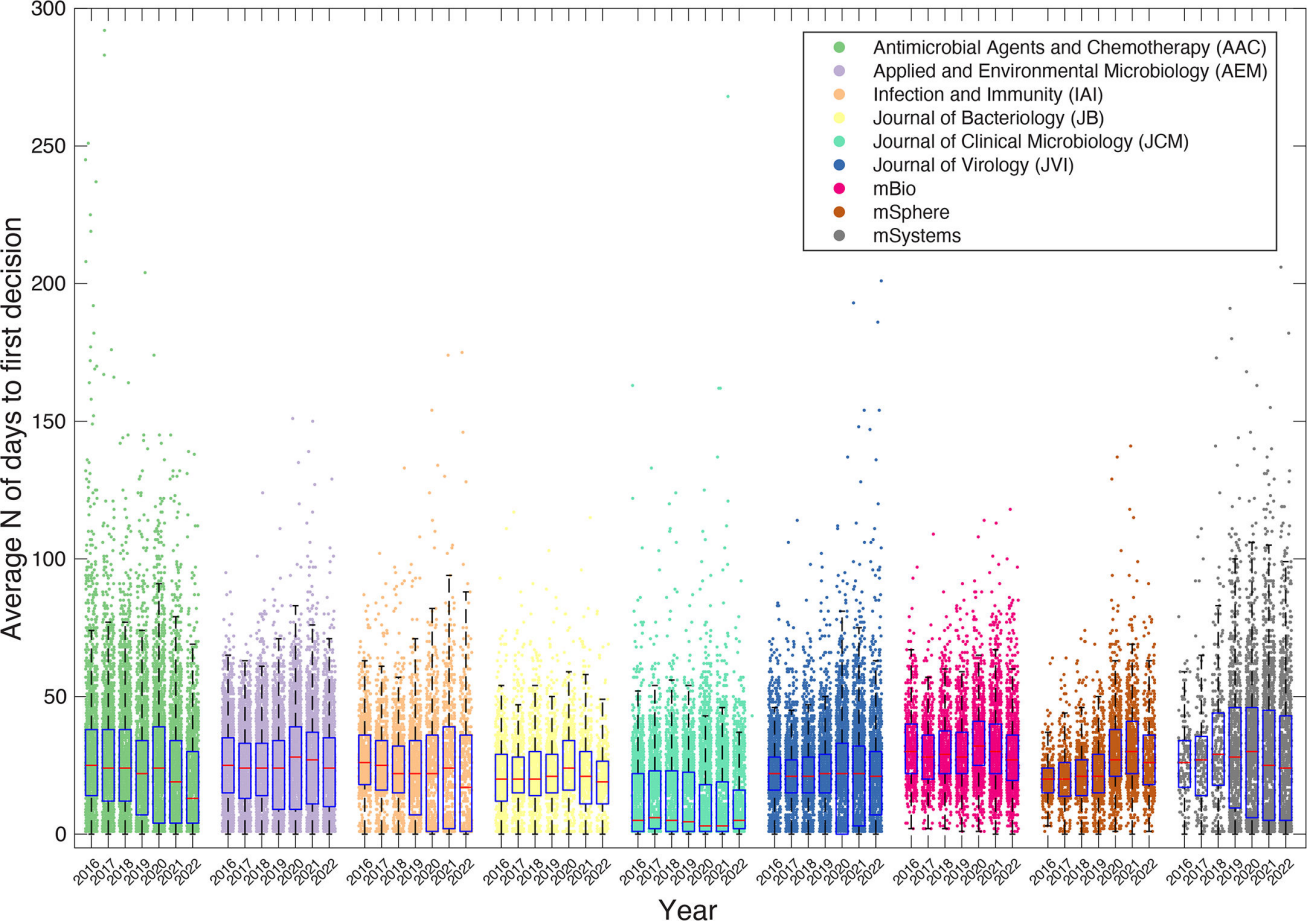
The average number of days from receipt to first decision (this includes the time from completion of the final review until the editor sent the decision letter) that a submission spent in peer review prior to publication broken out across the nine ASM journals. Error bars indicate standard error.

Interestingly, we found that between 2016 and 2019 across these nine ASM journals, the average time that a submission spent in peer review was relatively stable at between 26 and 27 days (Fig. 1). However, in 2020, time in peer review increased to ~31 days and has subsequently remained stable. When we break this down by journal (Fig. 2), the trend is variable. For example, the *Journal of Bacteriology* (JB) and the *Journal of Clinical Microbiology* (JCM) barely show a pandemic-associated increase in 2020; in fact, JCM appears to show a decrease in 2020 and 2021. In addition, while many journals show a 2020 increase and a resulting decrease back to the pre-pandemic levels, *Antimicrobial Agents and Chemotherapy* (AAC), *Infection and Immunity* (IAI), and *mSystems* all show lower times to first decision in 2022 and 2023 than they did before the pandemic, which is testament to the dedication to the peer review process by the reviewers, editors, and staff at these ASM journals. Therefore, while the average increase of around 4–5 days could potentially be associated with the onset of the pandemic and the much-reported “burnout phase” in 2020, the trend is not universal across journals.

**Fig 3:**
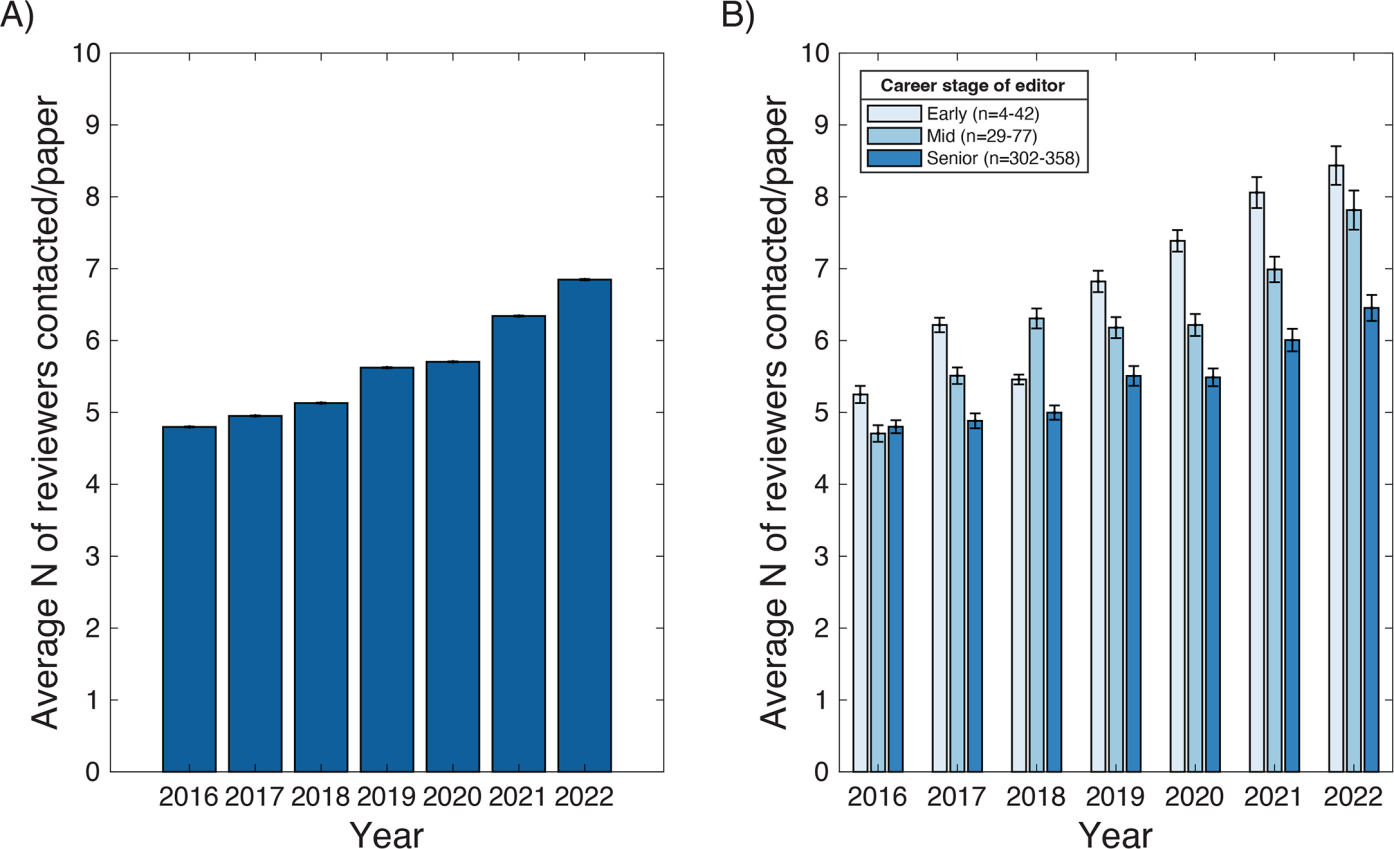
(A) The number of reviewers *N* that are contacted per article to be reviewed is increasing. (B) Editors of all career stages, early (postdoctoral or assistant professor), mid (associate professor), and senior (full professor and emeritus) are having to ask more people to review papers. Data from nine ASM journals. Error bars represent standard error. In the legend, *n* represents the number of editors represented in these categories between the years 2016 and 2022.

**Fig 4:**
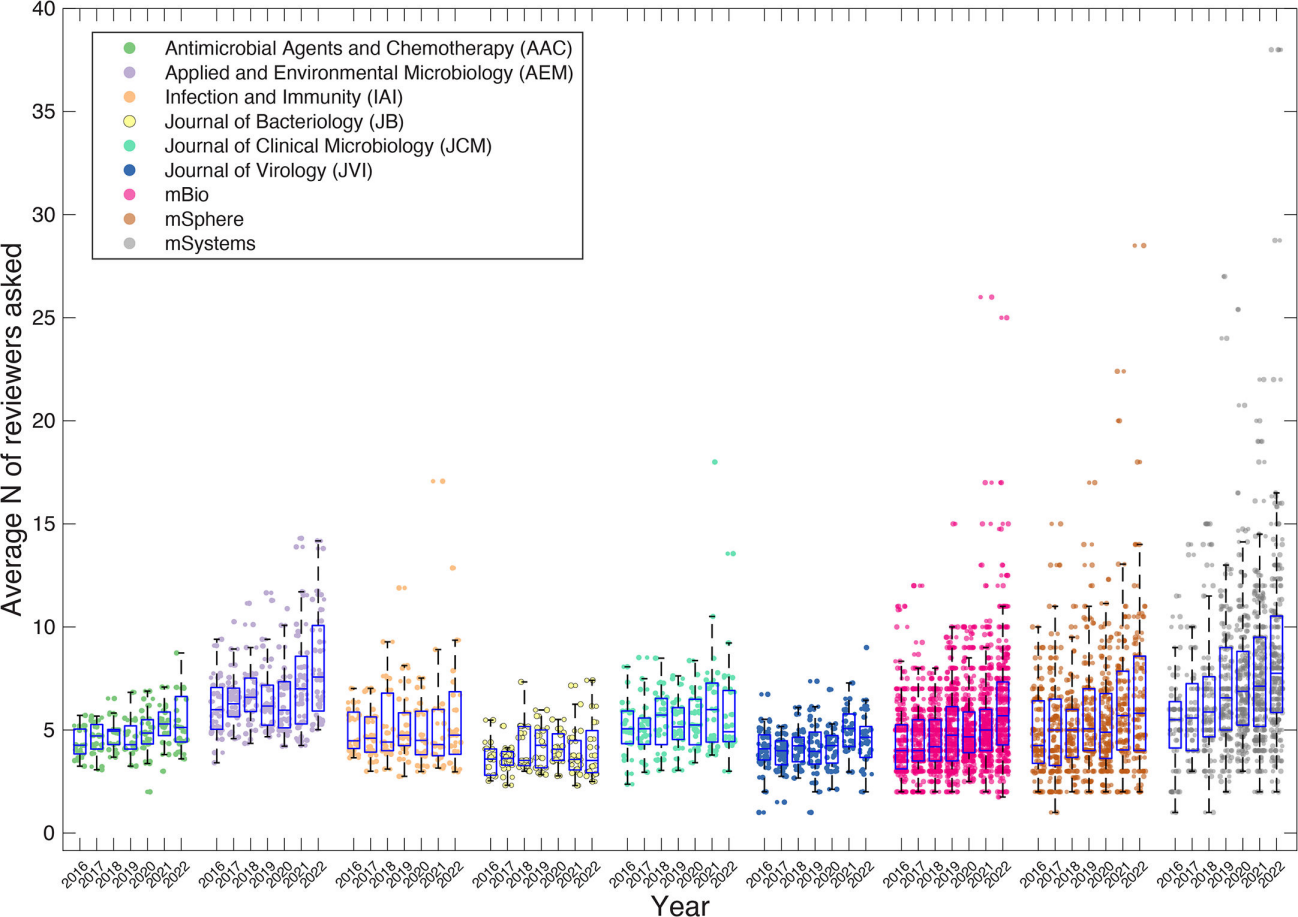
The number of reviewers *N* that are contacted per article to be reviewed across the nine different ASM journals.

We next asked whether the increase observed in 2020 in the time submissions spent in peer review was associated with an increase in the number of people sought to perform peer review. Strikingly, the average number of reviewers contacted across nine ASM journals increased by over 1.4-fold from 4.8 in 2016 to 6.8 in 2022 (Fig. 3A); however, this increase appeared to be a steady progression, rather than a dramatic shift associated with the pandemic, which is suggestive of two separate drivers. Importantly, the same increase is observed across six of the nine journals (Fig. 4), suggesting a more universal trend. The three journals that break from this trend are JB, JCM, and *Journal of Virology* (JVI), which show considerable variability over the observed years. These outliers are all at the lower end of “contacted reviewers,” and are among the quickest to first response, which shows a potential association between these two metrics. Examining how variable editorial practices across the journals may be contributing to the observed variance will require considerably more resources and data to determine.

We also posited that the career stage of the editor requesting peer review may influence whether a reviewer is likely to respond positively to a peer review request. This hypothesis was predicated on the assumption that the seniority and reputation of an editor in a field may influence response rates to their requests, either as a direct function of their “renown” or as a function of their knowledge of the field and of who may be more responsive to the request. While the number of reviewers contacted per article increased irrespective of editor’s career stage, early career editors consistently had to ask more reviewers than mid- and senior-stage editors (Fig. 3B). Furthermore, while in 2016 the difference in number of reviewers asked was less than 10%, by 2022 early career editors had to ask 30% more reviewers than senior editors, suggesting a progressively widening gap.

In summary, while the observed increase in time taken to review can potentially be explained by pandemic-associated “burn-out,” the steady increase in the number of people contacted to review points to a potentially bigger problem. It will be necessary to get data from many more journals to determine whether this trend is universal. Furthermore, while an increase in time taken to review is concerning for all microbiologists, it may have a bigger impact on early career scientists, especially when considering the myriad ways that early career scientists are disadvantaged. At the highest risk are underrepresented minorities who already face even more profound challenges within the academic system. While pre-prints are increasingly being considered as evidence of productivity, they lack the robust credibility of a peer-reviewed article, and so in the absence of an official acceptance at a peer-reviewed journal, they remain undervalued. Therefore, new solutions are necessary.

At a basic level, scientific reviewing should provide some kind of gratification to the reviewer, whether intellectual (staying at the forefront of the field), social (networking), cultural (status from being a respected reviewer), or monetary. Unfortunately, the current incentives to review are clearly not enough. Without any kind of credit, it becomes hard to justify spending time and energy reviewing when researchers are already overwhelmed. We posit that this exhaustion and frustration have led to a global decrease in the sense of belonging to a scientific community that requires altruism to function. We would like to encourage social scientists to engage in this perceived problem, to generate the data to test this proposition. However, in the absence of such data, where do we go from here? We think there are at minimum four immediate solutions: (i) increase the pool of reviewers, (ii) reward reviewing at the institutional level, (iii) institute journal-initiated reviewer rewards, and (iv) increase the use of artificial intelligence (AI).

(i) Increase the pool of reviewers. In a rapidly growing field of scientific inquiry, we often end up with a pyramid of researchers. This results in the majority being early career researchers (ECRs), such as graduate students, postdocs, and new assistant professors or equivalents. When people recommend reviewers for their submissions they often recommend “well-known” more senior career-stage individuals, which results in the bombardment of a few people, and hence most requests are declined. One possible solution to increase the reviewer pool is therefore to formally train ECRs in rigorous peer review and formally invite them to perform peer review (6, 7). This formal recognition of the valuable input of ECRs to peer review is being implemented in many academic journal settings (8), including at the American Society for Microbiology, which has an active ECR training program and resulting pool of talented peer reviewers that significantly increases the probability that editors will find a peer reviewer for a submission. This mechanism is currently only in place at *mBio*, and while the resulting pool of trained reviewers can be used by all ASM journals, the training mechanism could be implemented more widely. Outside of these initiatives, a practical challenge to implementing trainees as reviewers is that editors currently cannot easily access contacts of anyone but the corresponding authors—adding email information for all authors to the published paper could help provide more reviewers that are knowledgeable on the topic while improving the recognition of ECR co-authors. While we acknowledge that many ASM journals do acknowledge reviewers annually through publication (1) and/or awards (9), this practice could be more broadly implemented and receive more rigorous acknowledgment (see next section). Furthermore, new initiatives such as Review Commons and eLife are experimenting with new ways of doing more open peer reviews outside of the usual journal structure. More large-scale experiments of different reviewing models will enable new solutions to be created.

(ii) Reward reviewing at the institutional level. Academic institutions should promote reviewing and include this work as part of expected academic community service. Importantly, some fields are now recording peer review efforts through services such as Publons or ORCID. Instead of expecting peer review service as part of a career progression review, we recommend that some attempts be made to quantify the impact of an individual’s peer review activity and that this activity be associated with a rigorous assessment to demonstrate an individual’s impact through service on the community. This would raise the value of peer review activity within institutions. As an essential form of being a good science citizen, reviewing should be commensurate with publishing. To further facilitate this, journals could also be involved in providing standardized feedback and incentives to reviewers that produce quality reviews, which could be presented at promotion as evidence of impact.

(iii) Institute journal-initiated reviewer rewards. Another important improvement to peer review would be for journals to create an overall better experience for reviewers. This would start from having better triaging at the editorial stage, with friendlier online platforms, and more flexible policies. To improve triage, journals could require authors to become peer reviewers for articles submitted to the journal before they too would be able to submit their own work. This could help authors assess the fit of their article, as well as align their submission better to the journal itself. However, this effort is often not promoted in journals with limited impact due to the extra effort and complex assessment that is needed. Beyond the initial assessment by the authors and editors, the interactions between reviewers and the journal could be improved. Many journals have short turnaround times for reviewers, rely on clunky websites, and provide no perks for reviewers. While it is important to provide feedback to the authors quickly, a non-binary response (beyond accepting or declining to review) may prove useful—a reviewer could request a specific timeline for providing a review, which would allow the editor to agree, or seek further reviewers. Furthermore, providing a user-friendly web portal with clear instructions, more flexible timelines, and incentives for peer review would go a long way in encouraging reviewing and clearing the backlog. While financially compensating reviewers through an honorarium or a publishing credit will increase the already high publishing costs, it should not be dismissed out of hand as a potential solution. Trying out the policy of financial compensation for peer review in a series of journals would help to determine if any of the projected concerns are factual. One way to alleviate the high open access charges or color figure charges associated with publishing (as has already been done at some journals, including all ASM journals), is to improve institutional subsidies through universities or private organizations. Paid peer reviewers would also require editors to critically evaluate and only incentivize good reviewers such that there would be reduced risks of reviewers providing sub-par evaluations for personal gain. Aside from paying direct honoraria, another option to remunerate reviewers indirectly would be to provide their institution with publishing credits (as often happens at some journals for individual reviewers), which reduces future publishing costs for the institution. These credits could significantly benefit underrepresented minorities or scientists in low- and middle-income countries.

(iv) Increase the use of AI. While AI has already been used for identifying plagiarism and image falsification, it could be deployed further to reduce the number of more cumbersome reviewing tasks such as checking for the use of correct statistical tests, the correct use of the language and grammar (although this is often not asked of reviewer’s at many journals, and could raise concerns over authorship), that the appropriate references are listed, that reagents have not been discontinued, or that the figures are cited correctly. This could be done ahead of sending out the manuscript to reviewers, allowing them to obtain a pre-screened manuscript. Such a practice, while not without pitfalls, would reduce the overhead of the review process, and entrust reviewers specifically with the more focused task of judging whether the science presented is appropriate and a significant advancement.

The overburdening of academic service has widespread effects—the pandemic-associated increase in “time to publish,” compounded by the steady increase in the number of reviewers contacted are only symptoms of the problem—and may hide further downstream consequences, but one that will have further negative impacts. Good scholarship relies on a working, connected, and timely way of providing feedback and ensuring published articles are rigorous and accurate. It is time to revamp the current peer review system to ensure that trainees, scientists, and science as a whole are not irreversibly hindered.
